# American college of radiology ovarian-adnexal reporting and data system ultrasound (O-RADS): Diagnostic performance and inter-reviewer agreement for ovarian masses in children

**DOI:** 10.3389/fped.2023.1091735

**Published:** 2023-03-08

**Authors:** Huimin Wang, Limin Wang, Siwei An, Qiuping Ma, Yanping Tu, Ning Shang, Yunxiang Pan

**Affiliations:** Department of Ultrasound, Guangdong Women and Children Hospital, Guangzhou, China

**Keywords:** O-RADS, ovarian masses, ultrasound, diagnostic performance, children

## Abstract

**Objective:**

To evaluate the diagnostic performance and inter-observer agreement of the American College of Radiology Ovarian-Adnexal Reporting and Data System Ultrasound (O-RADS) in the diagnosis of ovarian masses in children.

**Methods:**

From June 2012 to December 2021, 163 ovarian masses in 159 patients with pathologic results were retrospectively analyzed. Each mass was classified into an O-RADS category according to the criteria. The diagnostic performance of O-RADS for detecting malignant ovarian masses was assessed using histopathology as the reference standard. Kappa (*k*) statistic was used to assess inter-observer agreement between a less-experienced and a well-experienced radiologist.

**Results:**

Out of 163 ovarian masses, 18 (11.0%) were malignant and 145 (89.0%) were benign. The malignancy rates of O-RADS 5, O-RADS 4, and O-RADS 3 masses were 72.7%, 34.6%, and 4.8%, respectively. The area under the receiver operating characteristic curve was 0.944 (95% CI, 0.908–0.981). The optimal cutoff value for predicting malignant ovarian masses was  > O-RADS 3 with a sensitivity, specificity, and accuracy of 94.4%, 86.2% and 86.2% respectively. The inter-observer agreement of the O-RADS category was good (*k *= 0.777).

**Conclusions:**

O-RADS has a high diagnostic performance for children with ovarian masses. It provides an effective malignant risk classification for ovarian masses in children, which shows high consistency between radiologists with different levels of experience.

## Introduction

Ovarian mass is a common disease in women, but its incidence in children is approximately 2.2–2.6 per 100,000 (1). The pathological type of ovarian mass in children is complex, most of which are benign masses, while malignant tumors account for approximately 4%–22% (1). Different from adult ovarian tumors that are mainly epithelial tumors, pediatric ovarian tumors are mainly derived from germ cell tumors. Most pediatric ovarian tumors (including malignant tumors) have a relatively good prognosis if treated timely, and the 5–10-year survival rates can reach 80%–90% (2). However, with the improvement of survival rate, the requirement of fertility preservation is a key consideration that poses a challenge to the choice of surgical method for ovarian masses in children. In this setting, surgeons need to balance the need for fertility preservation with that that of accurate staging and evaluation of the resection range of malignant tumors (3, 4). Moreover, avoiding resection of ovaries with benign tumors reduces the risk of premature menopause and its short and long-term sequelae such as infertility, osteoporosis, cardiovascular disease, and neurocognitive effects (5, 6). Preoperative assessment of the risk of malignancy for ovarian masses is a key imperative in order to strike a balance between fertility preservation and more aggressive cancer treatment (7, 8). Use of ultrasound for the differential diagnosis of benign and malignant ovarian masses in children is mainly based on the size and physical properties of the masses, but there are obvious limitations (8–10).

Structured reporting of the ultrasound findings of ovarian masses was identified by a consensus working group of a Society of Radiologists in Ultrasound as a key step for improving the management of women with ovarian masses (11). The structured reporting systems mainly include ovarian-adnexal reporting and data system (O-RADS), gynecologic imaging reporting and data system (GI-RADS), International Ovarian Tumor Analysis (IOTA) “Simple Rules” and “ADNEX” models. These models have shown a high diagnostic performance for women with ovarian masses (12–17). However, application of these models to pediatric ovarian tumors has not been reported. Therefore, the purpose of this study was to evaluate the diagnostic performance of ultrasound O-RADS in the differential diagnosis of benign and malignant ovarian tumors in children, so as to identify a more objective and standardized method for the preoperative evaluation of ovarian tumors in children.

## Materials and methods

### Population

We retrospectively analyzed children with ovarian masses confirmed by histopathological examination of surgical specimens at the Guangdong Women and Children Hospital between June 2012 and December 2021. Data pertaining to demographic characteristics, clinical examinations, pathologic diagnosis, surgical findings, and follow-up data were retrieved from the electronic medical case records. Inclusion criteria were: (a) age <18 years; (b) ovarian mass was detected by ultrasonography, and surgical treatment was performed to obtain clear pathological results; (c) ultrasound images were complete and clear. Exclusion criterion: histological findings were obtained more than 120 days after the ultrasound examination. Finally, 159 children were enrolled in this study.

### Examination methods

Ultrasound examinations were performed using high-resolution color Doppler ultrasound diagnostic apparatus such as Samsung (WS80A, RS80A), Aloka (*α*10), GE (VOLUSON E8, VOLUSON E6), Hitachi (HIVISON Preirus, 60/70), and Mindray (DC-8, Kunlun 7). The frequency of convex array probe was 2–8 MHz, the frequency of linear array probe was 4–12 MHz, and the frequency of intracavity probe was 5–10 MHz. Routine abdominal examination was performed. The bladder was moderately filled before examination. Patients were placed in a supine position to fully expose the lower abdomen, and the pelvic and abdominal cavity (if necessary) were comprehensively scanned. The size of the uterus and bilateral ovaries, and presence of any ovarian or pelvic mass was recorded. The size, shape, boundary, relationship with surrounding tissues, internal echo and blood flow of the tumor were recorded. When necessary, trans-rectal ultrasound examination was also performed for differential diagnosis. Written informed consent was obtained from a parent or guardian and the examination was performed in the presence of a parent or guardian. The imaging data of all cases were stored in Picture Archiving and Communication System (PACS) for analysis. All patients were followed up after surgery, and the results were confirmed by histopathological examination of surgical specimens.

### Retrospective images analysis

Ultrasound images were retrieved from the PACS. Before study set up, a resident radiologist with 3 years of experience learned the theory of the O-RADS lexicon and Risk Stratification and Management System. O-RADS classification of ultrasound images was performed by the resident radiologist, who was blinded to the clinical information and pathologic results. The radiologist described the ultrasound features and assigned an O-RADS category for each mass.

The O-RADS categories are (18): O-RADS 0: incomplete evaluation; O-RADS 1: definitively benign. Normal ovaries; O-RADS 2: almost certainly benign category (<1% risk of malignancy); O-RADS 3: low-risk category (1% to <10% risk of malignancy); O-RADS 4 intermediate-risk category (10% to <50% risk of malignancy); O-RADS 5: high-risk category (>50% risk of malignancy).

To assess inter-observer agreement with respect to O-RADS categorization between radiologists with different levels of experience, another radiologist with 9 years of experience performed a separate analysis for all the masses. The radiologist described the ultrasound features and performed O-RADS classification of the masses.

### Reference standard

The reference standard was histological diagnosis based on surgical specimen. Histopathology of masses were classified by the World Health Organization International Classification of Ovarian Tumors (19). As the same surgical intervention is recommended for borderline and malignant ovarian masses, borderline masses were defined as malignant (20).

### Statistical analysis

Data analyses were performed using SPSS version 20.0. Categorical variables were compared using the Chi-squared test. Non-normally distributed continuous variables were presented as median and inter-quartile range, and between-group differences were assessed using Mann–Whitney *U* test. Receiver operating characteristic (ROC) curve analysis was performed to calculate the areas under the curve (AUC) and determine the optimal cut-off values. Two-tailed *P*-values <0.05 were considered indicative of statistical significance.

We used Kappa (*k*) statistics to assess inter-observer agreement of ultrasound features and O-RADS category. The *k* values were interpreted as follows: poor agreement = 0.01–0.20; fair agreement = 0.21–0.40; moderate agreement = 0.41–0.60; good agreement = 0.61–0.80; very good agreement = 0.81–1.0.

## Results

### Patients and ovarian masses

A total of 163 ovarian masses in 159 patients were included in this study. The median age of patients was 13.0 (3.0, 16.0) years (range, 0–17). The detailed age distribution is shown in Table 1. There were 4 bilateral ovarian masses (all benign) and 155 unilateral ovarian masses. 145 (89.0%) masses were benign and 18 (11.0%) masses were malignant proven by pathology. Benign masses were mainly mature teratoma (*N* = 78, 53.8%), while malignant masses were mainly germ cell tumors (*N* = 9, 50.0%). The median age of the malignant group was 14.0 (11.8, 16.0) years, and that of the benign group was 13.0 (2.5, 15.0) years. The difference was not statistically significant (*P *= 0.052). The maximum median diameter of the tumor in the malignant group was 11.5 (7.3, 13.4) cm, which was significantly higher than that in the benign group [6.5 (4.9, 9.4) cm; *P *= 0.012].

**Table 1:** Age distribution of the patients.

Age (year)	Numbers(%)
<1	30 (18.4)
1–3	12 (7.4)
4–6	6 (3.7)
7–9	5 (3.1)
10–12	25 (15.3)
13–15	42 (25.8)
16–17	43 (26.4)

### Ultrasound features

The ultrasonic characteristics of benign and malignant ovarian tumors are compared (*χ*^2^ test) in Table 2. There was a significant difference between masses categorized as benign and malignant with respect to maximum diameter of masses, external contour, color score, and ascites (*P *< 0.05), which are the key terms in the O-RADS ultrasound lexicon.

**Table 2:** Ultrasound characteristics of ovarian masses.

Ultrasonic characteristics		Final diagnosis		*χ*^2^ test
		Benign (*n* = 145)	Malignant (*n* = 18)	*P*-value
Lesion category	Unilocular, no solid component	40	0	<0.001
	Unilocular cyst with solid component (s)	45	1	
	Multilocular cyst, no solid elements	22	0	
	Multilocular cyst with solid component (s)	35	10	
	Solid or solid appearing	3	7	
Maximum diameters of lesions (D)	D < 10 cm	114	7	<0.001
	D ≥ 10 cm	31	11	
Irregular external contour	Yes	5	6	<0.001
	No	140	12	
Color score	1	113	3	<0.001
	2	25	7	
	3	3	5	
	4	4	3	
Ascites	Yes	9	4	0.018
	No	136	14	

### O-RADS classification

A total of 163 masses were assessed. Of the 105 ovarian masses categorized as O-RADS 2, none was malignant; of the 21 ovarian masses categorized as O-RADS 3, one ovarian mass was malignant; of the 26 ovarian masses categorized as O-RADS 4, nine ovarian masses were malignant; and of the 11 ovarian masses categorized as O-RADS 5, eight were malignant. Table 3 summarizes the O-RADS classification and histological diagnosis of the ovarian masses.

**Table 3:** O-RADS classification and histological diagnosis of 163 ovarian masses.

Histologic diagnosis	O-RADS 2	O-RADS 3	O-RADS 4	O-RADS 5	Total
**Benign adnexal masses**	105	20	17	3	145
Mature teratoma	60	11	6	1	78
Follicular cyst	25	2	2	0	29
Serous cystadenoma	4	0	1	0	5
Mucinous cystadenoma	13	6	5	0	24
Corpus luteum	2	0	0	0	2
Endometrioma	0	0	1	0	1
Ovarian Theca-fibroma	0	0	1	2	3
Other benign adnexal masses	1	1	1	0	3
**Malignant adnexal masses**	0	1	9	8	18
Germ cell tumor	0	0	3	6	9
Sex cord-stromal tumor	0	1	3	2	6
Borderline tumor	0	0	3	0	3
Total	105	21	26	11	163

### Diagnostic performance

**Figure 1:**
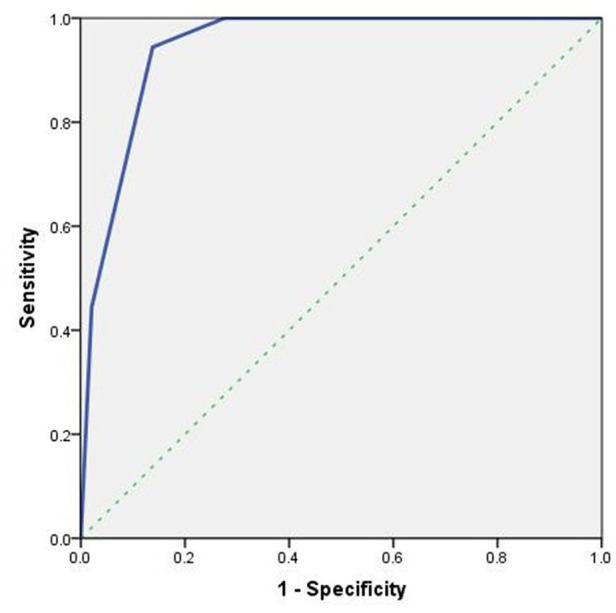
Receiver operating characteristic (ROC) curve analysis of O-RADS.

The malignancy rates of O-RADS 5, O-RADS 4 and O-RADS 3 lesions were 72.7%, 34.6%, and 4.8% respectively. For O-RADS classification, the area under the ROC curve was 0.944 (95% CI, 0.908–0.981) and the optimal cutoff value for predicting malignant ovarian masses was > O-RADS 3 (Figure 1). The sensitivity, specificity, accuracy, positive predictive value, and negative predictive value of O-RADS 4 and 5 categorization for malignant lesions were 94.4%, 86.2%, 86.2%, 45.9%, and 99.2%, respectively.

The O-RADS 4 lesions include the following: (1) unilocular cyst with solid component; (2) multilocular cyst, no solid elements; (3) multilocular cyst with solid component; (4) solid or solid appearing. If unilocular cyst with solid component and multilocular cyst with no solid elements are categorized as O-RADS 4A masses and the remaining cystic lesions with solid components are categorized as O-RADS 4B masses, the malignancy rates were 10.0% and 50.0%, respectively (Table 4), which indicated significant improvement in risk stratification (*P *= 0.037).

**Table 4:** Malignant risk in sub-groups of O-RADS 4 masses.

	O-RADS 4A	O-RADS 4B	
Benign	9	8	17
Malignant	1	8	9
Total	10	16	26

**Figure 2:**
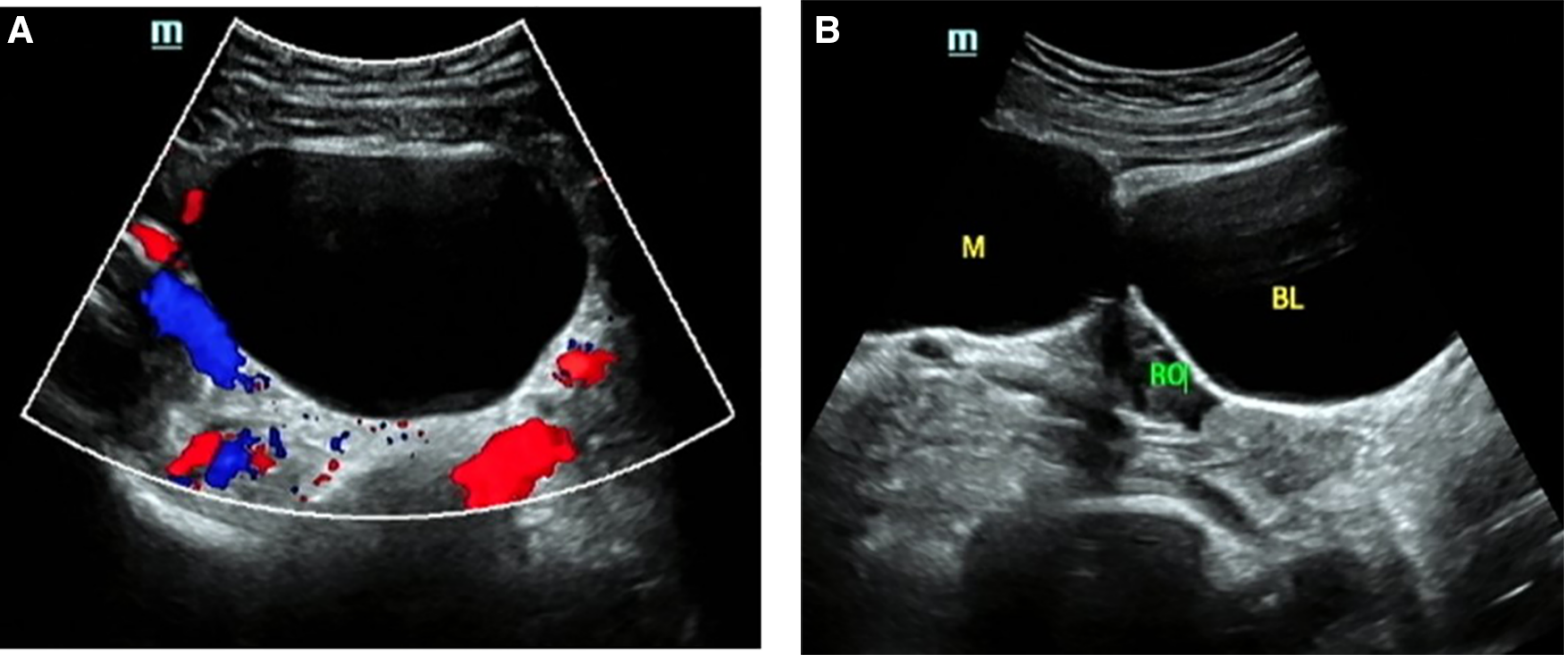
O-RADS 2, maximum diameters of lesion 79 mm, pathology: mature teratoma. (**A**) Unilocular cyst, no solid elements, color score 1; (**B**) The mass was located beside the right ovary. M, mass; RO, right ovary; BL, bladder.

**Figure 3:**
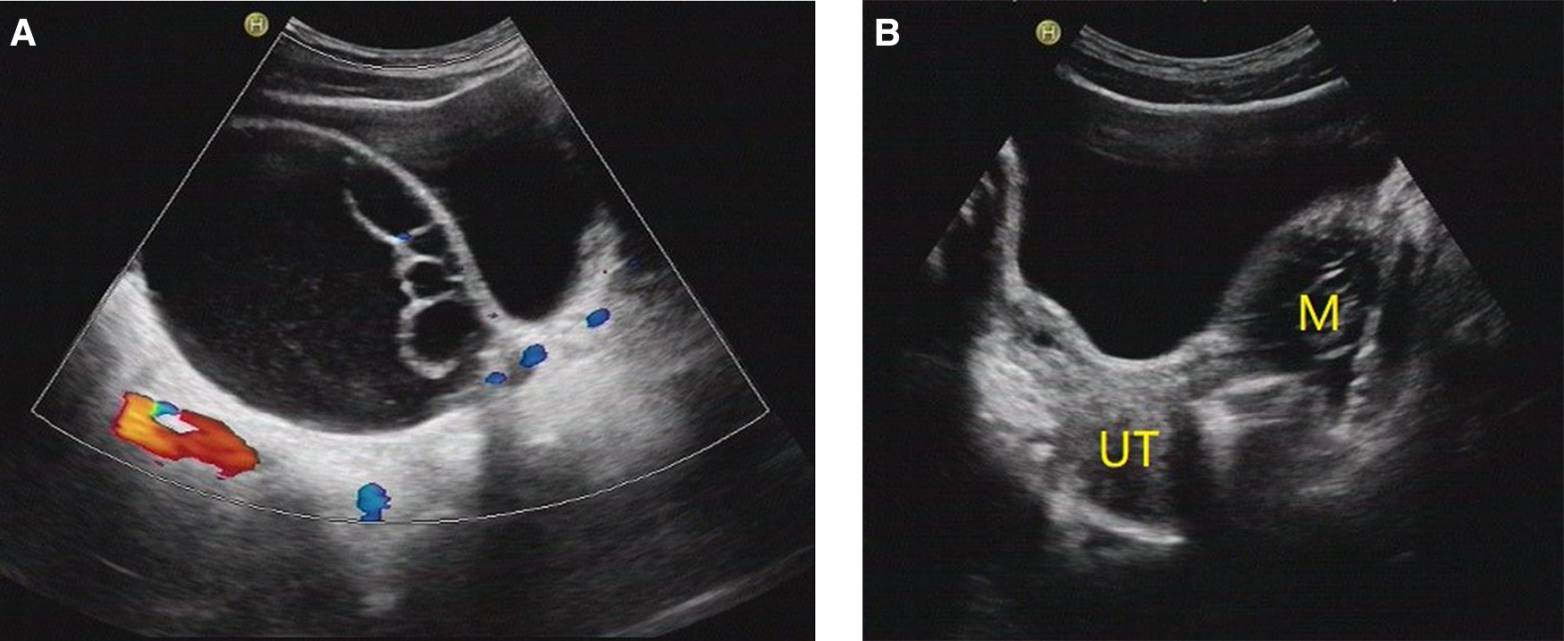
O-RADS 3, maximum diameters of lesion 93 mm, pathology: serous cystadenoma. (**A**) Multilocular cyst, no solid elements, color score 2; (**B**) The mass was located on the left side of the uterus. M, mass; UT, uterus.

**Figure 4:**
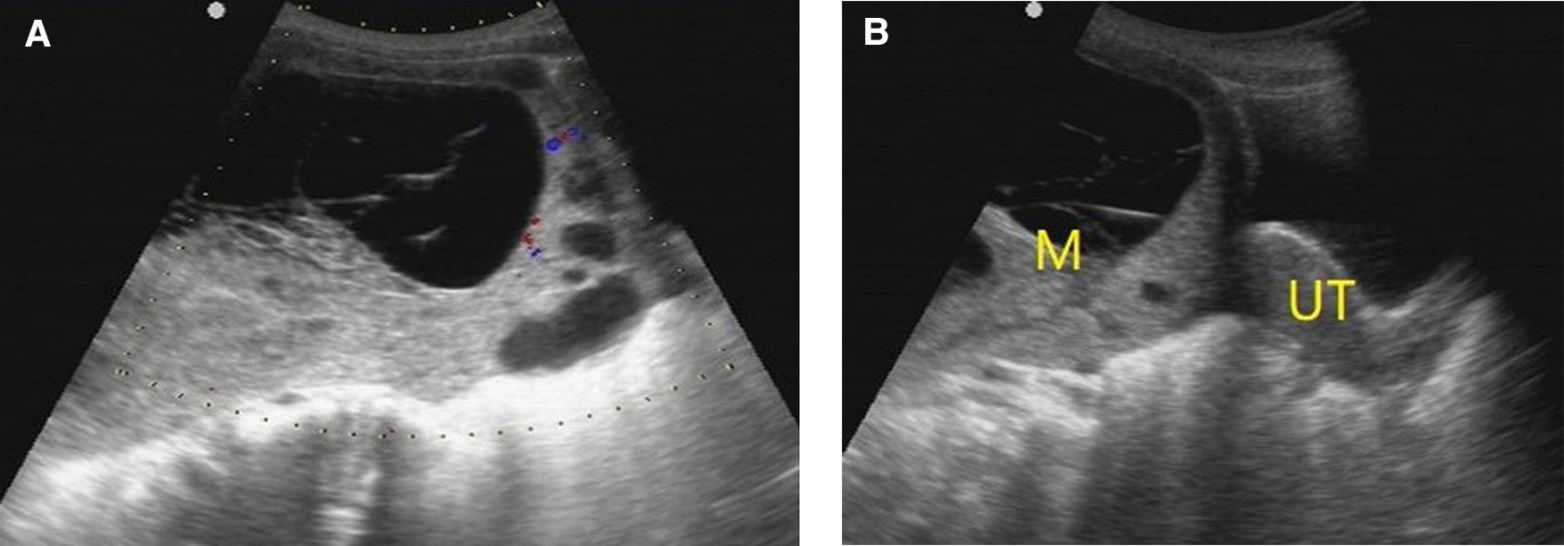
O-RADS 4, pathology: yolk sac tumor. (**A**) Multilocular cyst, solid elements, color score 2; (**B**) The mass was located above the uterus. M, mass; UT, uterus.

**Figure 5:**
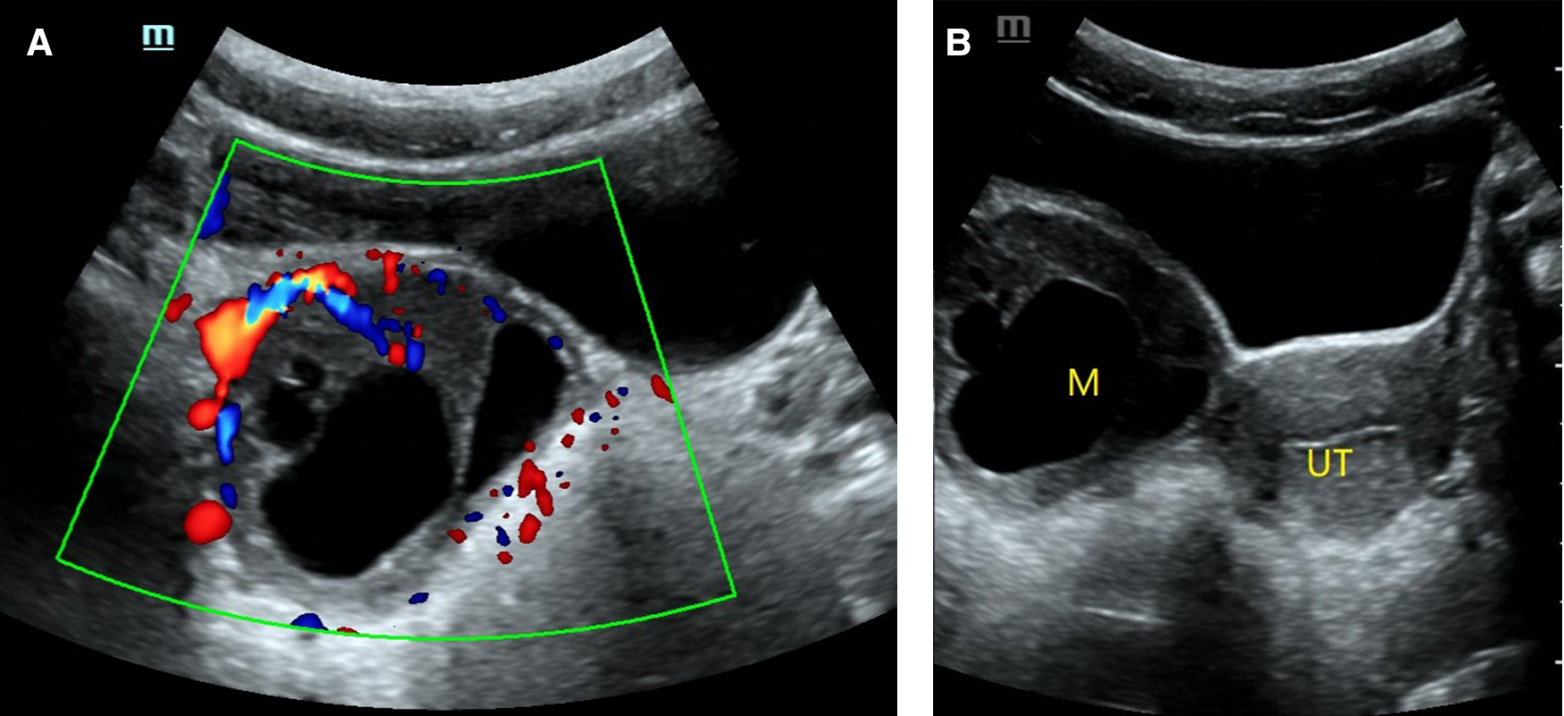
O-RADS 5, pathology: moderate differentiated Sertoli-leydig cell tumor. (**A**) Multilocular cyst, solid elements, color score 3; (**B**) The mass was located on the right side of the uterus. M, mass; UT, uterus.

Figures 2–5 show the ultrasound findings of O-RADS 2, 3, 4 and 5 masses.

### Inter-observer agreement between different levels radiologists

Inter-observer agreement between a radiologist with 3 years of experience (Observer 1) and a radiologist with 9 years of experience (Observer 2) was assessed regarding ultrasound features and O-RADS category. The inter-observer agreement of the O-RADS category was good (*k *= 0.777, *P *< 0.001) (Table 5). With respect to description of ultrasound features, we found very good inter-observer agreement respect to with identification of ascites (*k *= 0.853, *P *< 0.001) and classification of masses categories (*k *= 0.847, *P* < 0.001). The inter-observer agreement was good for color scores (*k *= 0.655, *P *< 0.001) and external contour (*k *= 0.681, *P *< 0.001).

**Table 5:** Inter-observer agreement of O-RADS classification.

		Observer 2				Total
		O-RADS 2	O-RADS 3	O-RADS 4	O-RADS 5	
Observer 1	O-RADS 2	92	9	3	1	105
	O-RADS 3	1	17	3	0	21
	O-RADS 4	0	0	22	4	26
	O-RADS 5	0	0	0	11	11
Total		93	26	28	16	163

## Discussion

In this study we evaluated the diagnostic performance and inter-observer agreement with respect to ACR O-RADS categorization of ovarian masses in a Chinese pediatric cohort. In addition, we verified the ultrasound risk stratification of O-RADS classification, and evaluated the differences between benign and malignant ovarian tumors with respect to the key terms in the dictionary. The results showed that the diagnostic performance of O-RADS for children with ovarian masses was good, and the inter-observer reliability among radiologists with different levels of experience was high. Our findings also suggest that O-RADS provide an effective risk stratification of malignant tumors for children ovarian masses, and the sub-classification of O-RADS4 masses can provide better risk stratification.

In our cohort, malignant tumors accounted for 11.0% of ovarian tumors which is consistent with a multi-center study by Madenci et al. (21). We also found that the maximum diameter of malignant tumors was significantly larger than that of benign tumors, which is consistent with that reported by Papic et al. and Lala et al. (10, 22). In 2020, ACR officially released a consensus guide for ultrasound risk stratification and management for O-RADS (18). The consensus guide is based on the O-RADS ultrasound dictionary published by the ACR ultrasound working group in 2018 (23). It is the only dictionary and risk-stratification system that contains all risk categories and related management schemes. Studies have shown that ultrasound O-RADS has good value in differentiating benign from malignant ovarian tumors in adult women (24).

Therefore, this study expounded the application value of O-RADS classification for pediatric ovarian masses from the aspects of inter-observer consistency, diagnostic threshold, and diagnostic performance, so as to provide an objective, reliable, and standardized classification method for the identification of benign and malignant ovarian masses in children.

In this study, we observed a significant difference benign and malignant tumors with respect to color Doppler score, presence of ascites, lesion type, lesion size, and external contour (*P *< 0.05), which are also the key terms in the O-RADS ultrasound lexicon. However, we note that not all terms in the lexicon are selected into the risk stratification system, such as acoustic shadowing. We found that 4 of 18 malignant masses had acoustic shadows (22.2%), and 46 of 145 benign masses had acoustic shadows (31.7%). Acoustic shadow may be a key feature to distinguish between benign and malignant tumors.

In our cohort, the number of cases with O-RADS1 class was 0, because all masses in our study were confirmed by surgery and pathological results, and O-RADS1 class indicates normal adnexa, which was not included in this study. The number of O-RADS 2 was the largest, because of the large proportion of benign masses in this group, and it was also consistent with the distribution of disease. In this study, the malignant rates of O-RADS in categories 2, 3, 4 and 5 were 0, 4.8%, 34.6% and 72.7%, respectively. Based on pathological results, the malignant rate of each O-RADS category was basically consistent with the risk recommended by the system (18). Cao L et al. also found a similar risk of malignancy for O-RADS 2 (0.45%), 3 (1.10%), 4 (34.46%), and 5 (89.57%) masses in adult patients with adnexal masses (24). Another study also showed a similar risk (2.8%) of malignancy for O-RADS 3 masses (25). The recommended risk of O-RADS 4 is between 10% and 50%, and in this study the malignant risk of O-RADS 4 was 34.6%. Therefore, it is still difficult to determine whether O-RADS 4 masses are malignant or benign. We tried to subdivide O-RADS 4 masses into two categories to obtain more accurate stratification. O-RADS 4A was associated with a malignant risk of 10%; For O-RADS 4B, the risk of malignancy was 50%. Therefore, the sub-classification of O-RADS4 masses can provide better risk stratification (*P *< 0.05). Therefore, we believe that it is very important to sub-classify O-RADS4.

In this study, the diagnostic threshold of ultrasound O-RADS classification for the differential diagnosis of benign and malignant ovarian masses in children was > O-RADS 3, which was consistent with the diagnostic threshold of O-RADS in the differential diagnosis of benign and malignant ovarian tumors in adults (24, 26). In this study, O-RADS 4–5 were diagnosed as malignant masses. The diagnostic performance of O-RADS classification for benign and malignant masses was very high (AUC: 0.944), indicating that O-RADS provides a good tool for differentiating benign and malignant ovarian masses in children, with high sensitivity (94.4%) and negative predictive value (99.2%). At the same time, the specificity of O-RADS classification for detecting malignant tumors in this study was 86.2%, and the positive predictive value was 45.9%, indicating that a large proportion of tumors diagnosed as malignant by O-RADS classification were benign tumors, which was mainly due to the fact that the ultrasonographic images of benign tumors such as mature teratoma, benign cystadenoma, and follicular membrane-fibroma may be characterized by multilocular tumors accompanied by malignant signs such as solid component, echo clutter, solid tumors, and slightly rich blood flow signals.Therefore, benign tumors are also likely to be classified into O-RADS 4–5 categories. For this subset of children, further differential diagnosis should be made based on clinical manifestations, laboratory tests (21, 27), MRI (28), and other imaging examinations.

It is very important to study the consistency of O-RADS classification results among different radiologists because O-RADS classification is based on ultrasound features which are liable to be influenced by subjectivity. Cao et al. (24) found good consistency between inexperienced radiologists and expert radiologists with respect to the description and classification of accessory lesions. This indicates that O-RADS has a good application for radiologists with different levels of experience. Pi et al. (26) reported that, even without specialized training, experienced ultrasound readers can achieve excellent diagnostic results and higher inter-reader reliability through self-study of guidelines and cases. So does O-RADS have good classification consistency in assessing the risk of malignant ovarian masses in children? This study found good consistency between radiologist with different experience levels with respect to O-RADS classification of pediatric ovarian masses (*k *= 0.777). Our results showed that the results of O-RADS classification may not rely on the work experience of ultrasound doctors, and to some extent, it reduces the diagnostic differences caused by subjective factors, and facilitates the communication between radiologists and clinicians. Our findings suggest that O-RADS classification is an objective classification method for the evaluation of ovarian masses in children, which is worthy of popularization and application.

However, this was a retrospective study of ultrasound images, which may have introduced an element of bias. Due to the low incidence and low malignant rate of ovarian masses in children, this study is based on a low number of tumors (163 benign and 18 malignant). In addition, in this retrospective study, it was not possible to identify the indications for surgery in patients with O-RADS 2 or 3 lesions. O-RADS also recommends close follow-up or management by gynecological experts for O-RADS 2 and O-RADS 3 masses. However, there are some limitations for O-RADS: Unlike the IOTA ADNEX model, O-RADS cannot provide individual risk of each lesion and is more cumbersome; And it needs to be emphasized that in this study the O-RADS is not a screening test but is used to attempt differentiating between benign and malignant tumors, once these tumors have been observed by ultrasound; O-RADS provides recommendations purely based on findings and often suggests unnecessary prolonged follow-up or additional testing; O-RADS may not be suitable for experts who always perform well, if not just checking images.

## Conclusions

In this study, O-RADS showed a high diagnostic performance for children with ovarian masses. Its high sensitivity and negative predictive value may help avoid missed diagnosis of ovarian malignant tumors in children, and provide the basis for timely intervention and preoperative evaluation. It provides an effective malignant risk classification for ovarian masses in children, which shows high consistency between radiologists with different levels of experience. In particular, this study found that the sub-classification of O-RADS4 masses can provide better risk stratification. Therefore, prospective, multicenter studies are required to provide more robust evidence of the diagnostic performance of O-RADS for pediatric masses.

## Data Availability

The original contributions presented in the study are included in the article/Supplementary Material, further inquiries can be directed to the corresponding author/s.
